# The Etiology and Treatment of Typhoid Fever

**Published:** 1861-05

**Authors:** V. H Taliaferro

**Affiliations:** Columbus, Georgia


### ARTICLE III.

*The Etiology and Treatment of Typhoid Fever.* By V. H. TALIAFERRO, M. D., of Columbus, Georgia.

Read before the Medical Association of the State of Georgia, April, 1861.

It is with much hesitation we appear before the profession, upon a subject of such paramount interest, and so obscure in its causes. We are encouraged, however, by the hope that, if no other good is accomplished, we may, at least, stimulate a more diligent and thorough investigation into the *obscure* features of typhoid fever. Some of the best talents in our profession have been devoted to the investigation of this subject, among whom we may mention as first and foremost among the French, Louis and Chomel; and your American physicians, Watson, Bartlett, Wood, Nathan Smith, Jackson, and others, all of whom agree upon the one important point, viz: "the nature and essence of the actual, producing, efficient cause of typhoid fever is entirely unknown." This general recognition and common consent, as to the obscurity of the *real cause* of typhoid fever, has unquestionably had the effect to paralyze the energies of the profession in the effort to bring this to light. Pathologists, who have been diligent and untiring in their developments, far from guiding aright the therapeutist, have but confounded and led into error. From time to time, the most opposite and hazardous plans of treatment have been adopted, all based upon the several views maintained, of lesions discovered. Louis, who has been regarded, since the publication of his elaborate work, as first in authority upon this subject, treated the disease by blood-letting, evacuants and tonics. Bouillard's method consisted in copious and frequently repeated abstractions of blood, in quantity, from twelve to sixteen ounces, and at the same time quite an equal amount being taken by means of leeches and cups. He, furthermore, proceeds to announce, "that success or cure is the law; failure or death, the exception." DeLarrogne's plan, which has been adopted to a very considerable extent by Andral, and others, was emeto-cathartic, and well nigh exclusively. Tartar emetic, calomel, castor oil, etc., were his remedies.

Dr. Nathan Smith says, that "he has seen many cases, in which persons in the early stages of the disease were moping about, not very sick, but far from being well, and who, upon taking a dose of tartrate of antimony, have been immediately confined to their beds."

These gentlemen were led to the adoption of their hazardous plans of treatment, by the development of lesions in the intestines, which were regarded as the unequivocal results of inflammation. And, indeed, with their views they could have adopted no other course. Many of us who have been following in the wake of these great minds, have been induced, we may say, from melancholy experience, to let our typhoid patients alone, and give nature an opportunity to effect her work of reparation. The many who, in latter days, have adopted this plan, and forsaken those lessons imbibed in error, can announce with far more truth than himself, the words of Bouillard: "that success or cure is the law; failure or death, the exception."

Now it is the purpose of this essay to establish by facts, based upon physiology and pathology, the essential and legitimate cause of typhoid fever:

1st, It is claimed that the primary disturbance is in the physiological functions of the skin. 2d, That by this disturbance the *excrements* of the cutaneous system are retained in the blood, and from the action of which excrements begins, in fact, the morbid process in the vital functions of that fluid, which gives existence and development to the peculiar and characteristic phenomena of this disease.

Dr. O. C. Gibbs, who prepares a valuable and highly interesting summary in the *American Medical Monthly*, urges the following objections to this theory, which was first advanced by me and published in the *Atlanta Medical and Surgical Journal:*

1st, "If the theory is correct, in all cases where the perspiratory secretions are arrested or materially suppressed, we should get a typhoid fever." 2d, "We have no evidence that a disturbance of the exhaling functions of the skin precedes the febrile action in any considerable number of cases of typhoid fever." 3d, "If a suppression of the function of the skin is the primary pathological condition in this disease, as the sympathetic connection between the skin on the one hand, and the lungs and kidneys on the other is far more intimate than between the skin and bowels, the more constant pathological lesion should be found in the lungs or kidneys, and *not* in the glands of Peyer, as is well known to be the case." The Doctor thought, doubtless, to have completely demolished us with these three simple propositions, but we shall show most conclusively that they are of no weight whatever.

To the first objection I will say, that I did not and do not now hold, that all cases of suppression of the perspiratory secretion are followed by typhoid fever; but do contend that where the interference with the perspiratory function is of such character as to keep the blood constantly loaded with the cutaneous excrements, that it becomes materially and essentially changed, and that typhoid fever is the consequence. Furthermore, that this excrementitious poison acts as the *immediate* and *exciting* cause, while *many* circumstances may operate as great *predisposing* causes. The disease developed in most instances by the *exciting* agent, must depend in a great measure upon the peculiar predisposition at the same time existing. For example, malarial poison may develop other diseases than remittent fever; likewise a disturbance in the depurating function of the skin may cause other diseases than typhoid fever, as consumption and dyspepsia.

To the second objection, I will simply reply that we have the most conclusive and overwhelming evidence of *diseased skin* in typhoid fever, and that it cannot, possibly, ever fall to the lot of the physician to determine by *actual observation*, that this cutaneous disturbance *produces* this development in every case of the fever, from the fact that he is never called on for advice, until fever has set in, and his services considered requisite; but if he should find in the great majority of cases to which he is called, such a collection of cutaneous excrement upon the surface, as naturally and necessarily to interfere with its healthfulness, we must at least conclude that it is rather strong evidence in our favor. I hold that this condition of the skin, upon close examination, will be found in most cases of typhoid fever.

To the third objection, I affirm, as a physiological fact, that the relationship and sympathy between the cutaneous surface and that of the mucous membrane, is of far more intimate character, than the relationship and sympathy between the skin and kidneys. While it is true, that any sudden and violent impression made upon the cutaneous surface which checks its functions, is immediately perceptible in the increased action of the kidneys, it does not necessarily follow that it is the result of *sympathetic connection*, but more properly of a *vicarious* action; as the bleeding of the nose or of an ulcer in suppression of the menstrual function. The kidneys, for a time, may perform this vicarious action, but at length will become unable to perform their own individual function from the heavy tax of their over-burdened and double duty.

In this connection I will state, that it is usual, so far as my own observation extends, in the first stages of typhoid fever, for the kidneys to act more profusely than in health, showing that, from some cause or other, they are unusually excited. Other physicians have observed the same fact, and have expressed their astonishment at the occasional profuse action of the kidneys in this disease. In all other forms, the secretion of urine falls *far below* the natural quantity, not unfrequently being cut off two-thirds or more. Dr. J. S. Weatherly, of Montgomery, Ala., whose experience and observation in this disease has been quite extensive, in a letter of recent date, says: "In the great majority of cases of typhoid fever, I have found the kidneys acting almost naturally, as regards quantity, color, &c. Sometimes, however, I have found them, for the first few days of the attack, secreting large quantities of light colored urine; much more than in health. This condition of the kidneys, though, does not usually last throughout the attack; but towards the termination, particularly, if it be prolonged, and a severe case, the secretion becomes scant, and of a high color." This we regard as a strong link in the chain of facts which go to the making up, upon a sound basis, this cutaneous theory. In the commencement of the attack, we see the kidneys taking on this vicarious action, but being unable to perform their own with the double function of the skin, fall finally, from overwrought action, into a state of dangerous inactivity, leaving the blood in fact, no great unobstructed depurating channel but the lungs, and they, too, not unfrequently become the seat of serious lesion.

The functions of the skin may be recorded as follows: 1st, It is a great depurating organ. 2d, The prime regulator of the animal temperature. 3d, A blood aerating organ. According to the experiments of Seguin, and others, the amount of perspiratory fluid secreted daily, as insensible perspiration, is from two to three pounds. This may be largely increased from any circumstance calculated to stimulate the cutaneous function. Chemists have found the perspiration to contain soda, sulphur, potash, animal matter, acetic acid, butyric acid, formic acid, carbonic acid, and sometimes urea. It is estimated that the number of sudoriferous glands, which perform this important office of depuration, is about seven millions, and the total length of their tubing about 28 miles. The external coating of the skin is of a hard, horny and impermeable nature, for the necessary lubrication and softening of which, the sebaceous glands are abundantly distributed over the surface. But for this wise provision, we should, doubtless, have the skin, instead of as it is in its heathly state—soft and flexible—hard and unyielding, and completely barring up the mouths of the perspiratory vessels. We may arrive at the great and vital importance of the healthy and undisturbed function of these vessels, by the fact that impervious coatings to the entire surface of the skin, destroy life in an exceedingly short time, by the intensity of action of the retained perspiratory matter upon the blood. Now when we remember the fact that comparatively the fewest number of our ever busy people regularly wash their skins, and many not at all, can we conclude otherwise than that this neglect must result in mischief, of serious character, to the economy? And when washing the entire surface is resorted to, from the imperfect manner in which it is done, it amounts to but little or nothing. When the skin has been for a long time neglected, we find an immense accumulation of detritus and incrusted perspiratory matter, which must of necessity, materially disable the perspiratory vessels, as well as block up the sebaceous glands, thereby cutting off the supply of nature's oil to the skin, and forcing upon the blood a large amount of excrementitious elements which have served their purposes in the economy and are prepared for elimination.

The most important processes or functions in the human economy are those of depuration, by which the tissues and blood, and the entire economy are freed from disintegrated and noxious materials. These organs are the cutaneous system, the kidneys and the lungs. It will perhaps not be uninteresting to compare the analysis of these depurating elements:

| *Perspiration.* | *Urine.* | *Expired air.* |
|---|---|---|
| Water. | Water. | Water. |
| Animal extract. | Animal and mucus extractive matter. | Nitrogen. |
| Urea. | Urea. | Carbonic acid. |
| Sulphate of Soda. | Uric Acid. | |
| Salts of Potash. | Sulphate of Soda. | |
| Phosphate of Lime. | " " Potash. | |
| Carbonate of Soda. | " " Lime. | |
| Lactate of Ammonia. | Biphosphate of Soda. | |
| Chloride of Sodium. | " " Magnesia | |
| Fat. | " " Ammonia. | |
| Carbonic acid | Chloride of Sodium. | |
| Lactic Acid. | " " Potassium. | |
| Acetic Acid. | | |
| Butyric Acid. | | |
| Hydroclorate of Ammonia. | | |
| Phosphate of Ammonia. | | |
| Salts of Potash. | | |

Thus we find a striking analogy between these depurating fluids. Now, while it is true that the kidneys and skin may act vicariously, one with the other, for a limited time, it is evident that neither can habituate itself to the performance of a double office. The functions of the skin being depuratory, any interference with that function must, of necessity, result in mischief to the economy. What that mischief most frequently is, I think will be readily seen to be typhoid fever.

All excrementitious animal matter rapidly becomes putrescent, and it is known that putrescent matter, introduced into the blood, generates in it a like tendency to putrescence, by which all the tissues become impressed. One of the most destructive and alarming features of typhoid fever is its putrescent character. *This character may be developed by the retention in the blood of excrementitious animal matter.* Magendie confined a healthy dog in a situation which exposed him to putrid miasmata. For the first few days there was no change; he then began to emaciate, and died, much extenuated, within six days. Magendie imputed the death of the animal to the effect of the miasmata he respired and took with his food. On opening the body, which was much emaciated, *the mucous membrane of the bowels was found inflamed.* Now, the cutaneous excrements, when retained, become foreign and noxious to the blood, and assume, necessarily, a putrescent character. These facts settled, it next becomes necessary to show that in every case of typhoid fever the *skin is diseased.*

THE SKIN.

The convalescence of typhoid fever is invariably attended by a general *shedding of the cuticle and hair;* showing most conclusively that there must have been some diseased action in the skin. Furthermore, in the great majority of causes of typhoid fever I have seen examined with this view, the surface has been found incrusted with the debris of the skin, which may be easily ascertained by the forcible application of a little warm water and soap. The characteristic eruption of the skin in this disease is regarded as the result of an effort upon the part of the system to establish its depurating functions. Where there may have been doubts as to the diagnosis, it is always deemed satisfactory when the convalescence is attended with the pulling off of the cuticle and falling out of the hair. What has produced this condition of the skin, attended with so serious results? Usually a neglect of its cleanliness, and, in consequence, a vast accumulation of disintegrated perspiratory matter incrusted upon the surface, which must, of necessity, very materially obstruct the depuratory actions of the sudoriferous glands, by which the blood is poisoned, altered and disintegrated to such an extent as to lose its coagulating powers and its highly vitalizing properties, the ultimate results of which are to be found in the curious and distinctive phenomena of this remarkable disease. Here I would remark that other causes than the mechanical obstruction of the cutaneous vessels, may interrupt the depuratory actions of the skin and of course produce the same result. This interruption may be from an imperfect cappillary circulation or from other causes, but it is doubtless far more frequently the result of obstruction to the cutaneous vessels by a collection upon the surface of the perspiratory fluid and the debris of the skin.

THE BLOOD.

Pathology has thrown but little light upon the true character of the blood in typhoid fever. The most important results as yet ascertained, are, that there is a diminution of the fibrin, and of the force of aggregation, by which its particles are kept together. Indeed, the vitality of the blood seems, to a very considerable extent, lost. In the great majority of instances of *post mortems* in this disease, the heart has been found to contain dark uncoagulated blood. Bartlett says: "the blood drawn from the veins, during life, rarely exhibits the buffy coat; and when this is present, it is generally soft, gelatinous, or infiltrated and of a greyish or greenish color." This character of the blood has been particularly noticed by Louis, Chomel and Bouillard. It is affirmed by Bouillard that the blood of typhoid fever resembles that of no other disease. Upon this altered and vitiated state of the blood, evidently depends, the remarkably noxious and vitiated condition of all the secretions. The *odor* of the body is of a putrid, cadaverous character, and peculiar to typhoid fever. These facts taken together prove, beyond doubt, the putrescent character of the blood. Now, upon what depends this putrescence? Certainly upon the introduction into the blood of a *poison*. This poison cannot exist in malaria, for typhoid fever occurs oftenest in non-malarial districts; certainly not upon animal decay, or accumualation of filth, as was at one time supposed, for it is known to exist independent of any such thing. Indeed were it dependent upon an atmospheric poison, we should certainly have it occuring more or less as an endemic, which is far from being the case. It exists among all people and all classes and in all countries; upon the mountains and in the valleys. It occurs most frequently, and its fatality is greatest, where the atmosphere is regarded as purest, being exempt from all poisons, malarial or otherwise. This may be explained upon the principle that one poison may produce a *counter* impression upon the system to that of another, and hence the greater frequency of typhoid fever, where the atmosphere contains no counteracting agencies. *It occurs most frequent ly in Winter and Spring when the cutaneous system is more liable to interruption.*

RESPIRATORY APPARATUS.

Generally there are both bronchial and lung complications in typhoid fever, and sometimes of so serious a character as to threaten life, and lead to the diagnosis of *typhoid pneumonia*, as particularized by Prof. Dickson. The appearance of the diseased lung is wholly unlike the ordinary result of inflammation. Bartlett says: "It is quite unlike, in almost every respect, the second stage of inflammation, although the term *hepatization* has sometimes been applied to it." "It is not indicated by any peculiar symptoms during life." This condition of the lung (called by some *carnification*) is peculiar to typhoid fever. Upon no theory with which I am acquainted, can this condition be explained, save that of *cutaneous* obstruction; the morbid impression extending itself, by means of the intimate relationship and sympathy between the skin and lungs to the mucous membrane of the air passages. It may be contended that if it is the result of sympathy, it should present the characteristics of pneumonia. Doubtless it would, were the impressions upon the skin of sudden and violent character, as of cold, but the result must be very different when the skin has become diseased, by a longand continued accumulation of excrementitious matter, or other permanent interference with its depurating function, which has by slow degrees, well nigh, if not entirely cut off an important and highly essential depurating medium.

SMALL INTESTINES.

The characteristic lesion of the small intestines in typhoid fever, has given rise, very naturally, to a vast deal of discussion, vague surmises and learned essays, and is the basis in fact of the *French theory*, which, from the reputations and popularity of its illustrious advocate, Louis, has been widely popular in America, as in Europe. The great failure and hazardous success, however, which has attended the depletent and evacuant plans of treatment, founded upon this theory, appears to be rapidly (and fortunately for humanity) turning the current of opinion against it.

In the great majority of cases, the principle lesion consists in alterations of the elliptical plates or Peyer's glands, though not unfrequently there is *softening* of the mucous membrane of the small intestines, or of the entire tract, sometimes including the stomach, and which seems not to be the essential product of inflammation, as it exists most frequently without redness or thickening, and appears to be entirely independent of any inflammatory process or tendency, or other lesions of the intestines.

The more frequent lesion is, perhaps, in the *follicles* of the intestines, owing to the fact of their greater vitality, compared to the mucous membrane, and more frequently in the lower two-thirds of the illium, from the fact of the greatly increased number in that locality, and their arrangement into *groups* or *patches*. By very many pathologists, the lesion of these follicles is regarded as inflammatory, and as the prime seat and cause of the entire train of phenomena found in the disease. It would be somewhat out of place now to go into an elaborate argument to establish the fallacy and danger of such doctrine. Suffice it to say, the phenomena and physiological characters of the disease are *essentially unlike* the products and results of inflammation, even *redness* not being essential to the softening of the mucous membrane, so commonly found.

A very remarkable feature in the pathology of typhoid fever is *softennig* not alone of the mucous membrane, but of the spleen, the liver and the heart. Bartlett says: "the spleen is almost always altered in its appearance, the softening sometimes so extreme, that the parenchyma of the organ is reduced to almost an inorganic pulpy mass." Louis, furthermore, says: "that this softening is not the result of inflammation." This pathological change, then, must be the result of *loss of vitality*, which, of necessity, is followed by a disintegrating process, proportioned to the vital losses. These are the natural sequences of a retention in the blood of the cutaneous excrements.

The mucous membrane and follicles of the intestines become diseased in typhoid fever, from two great causes, viz: 1st, The circulation of an altered and vitiated blood. This fact was proven by the experiment of Magendie. 2d, By a protracted derangement of the cutaneous functions, owing to the intimate relationship and sympathy existing between the skin and mucous membrane.

The folicular groups or glands of Peyer, occupy most prominence, as the pathological seat of the disorder. Their function (so far as known) being the elimination of putrescent or noxious matter from the blood, their lesion in typhoid fever becomes a natural sequence, when considered in relation to this cutaneous theory. Dr. Reeves, whose valuable treatise upon enteric fever has become so vastly popular, says: "I choose to adopt that theory which supposes enteric fever to be the result of a specific poison, by some means introduced into the blood; that in the attempt to eliminate this poison from the blood-current, the glands of the bowels, whose office is assigned to be that of elimenating any putrescent accumulations from this fluid, become overburdened, and thenceforth result in more or less change of structure, that by such change the channels, also, through which nutriment reaches the blood, are more or less obstructed; that in consequence of this the blood becomes additionally depraved; and that these causes, primary and secondary, acting together, are capable of giving rise to the several conditions characteristic of enteric fever."

[TO BE CONCLUDED IN NEXT NUMBER.]

